# Current State-of-the-Art 3D MRI Sequences for Assessing Bone Morphology with Emphasis on Cranial and Spinal Imaging: A Narrative Review

**DOI:** 10.1055/a-2673-4339

**Published:** 2025-09-17

**Authors:** Irena Georgieva Kavrakova, Patrick Haage, Christoph Alexander Stueckle

**Affiliations:** 1 219505 Radiology, Niels-Stensen-Kliniken GmbH, Osnabrück, Germany; 2 12263 Humanmedizin, Universität Witten/Herdecke, Witten, Germany; 3 60865 Diagnostic and Interventional Radiology, HELIOS Universitatsklinikum Wuppertal, Wuppertal, Germany

**Keywords:** BlackBone MRI, ZTE, UTE, VIBE, FRACTURE, sCT

## Abstract

**Background:**

Traditionally, CT has been the go-to method for visualizing bone structures, while MRI has been preferred for assessing soft tissues, because structures containing tightly bound water molecules – such as bones, tendons, cartilage, and ligaments – produce a rapidly decaying T2* signal, which conventional MRI sequences fail to capture. To address this limitation, spoiled gradient echo sequences were refined, and short-TE sequences were introduced, enabling radiation-free bone imaging. This advance is particularly crucial for pediatric patients and in scenarios where an MRI-only approach is preferred, such as in radiation-sensitive cases and surgical planning.

**Methods:**

A comprehensive literature review was conducted by searching the PubMed and Google Scholar databases, using specific keywords: “black bone MRI” or “sCT bone” (Synthetic CT), “ZTE” (zero echo time), “UTE” (ultrashort echo time), “VIBE” (Volumetric Interpolated Breath-hold Examination), “FRACTURE” (FFE resembling a CT using restricted echo-spacing) and for title and abstract queries. The selection criteria included scientific articles published in English and German. The research was focused on the advances of the past five years in the application of the sequences in the area of the skull and spine. To support the technical understanding, earlier publications were also examined to offer readers essential background on the fundamental principles of the sequences, helping them better comprehend recent advances. For the investigation of the recent applications of the sequences, a narrow five-year time frame was applied, resulting in approximately 250 findings. From these, publications focused on the skull and spine regions were selected, with an emphasis on covering various pathologies and a preference for studies that compare different gradient echo sequences. To explore the technical aspects of the sequences, a broader time frame of ten years was selected, yielding approximately 868 results. From these, studies with more general explanations – avoiding in-depth physical and computer science details – were chosen. Using these selection parameters, 69 studies were highlighted.

**Results/conclusion:**

The gradient echo technique enables rapid and adaptable imaging, which can be customized to highlight specific tissue types. Spoiled GRE sequences such as VIBE, STAR/VIBE, and FRACTURE provide enhanced bone-to-soft tissue contrast, particularly when used with Dixon reconstruction. Short-TE sequences like UTE and ZTE utilize fast gradient switching, low flip angles, and non-Cartesian acquisition to improve bone visualization while suppressing soft tissue signals. These methods can effectively detect traumatic, neoplastic, and degenerative changes, offering CT-like imaging capabilities when patient-specific factors and the region or pathology of interest are properly considered. Additionally, integrating bone-selective sequences with deep learning could further enhance diagnostic accuracy and potentially replace CT.

**Key Points:**

- Short TE-sequences achieve better bone/soft tissue contrast, but are more computationally demanding.
- ZTE is the sequence of choice for skull vault pathology, preoperative spine and skull imaging and is a preferable base for neural networks in sCT generation.
- Modified UTE sequences excel in viscerocranium and spine imaging DURANDE for bone/air-interface, 3D-stack of stars UTE with Dixon reconstruction for spine pathologies, replacing the conventional MRI sequences.
- VIBE/STAR-VIBE for facial preoperative and traumatic imaging, where motion artifacts are problematic.
- Bone and ligament matrix quantification with Dual echo and IR-UTE-Cones sequence, emitting porosity index, suppression ratio, and mapping values.

**Citation Format:**

- Kavrakova IG, Haage P, Stueckle CA. Current State-of-the-Art 3D MRI Sequences for Assessing Bone Morphology with Emphasis on Cranial and Spinal Imaging: A Narrative Review. Rofo 2025; DOI 10.1055/a-2673-4339

## Zusammenfassung

**Hintergrund:**

Traditionell galt die CT als bevorzugte Methode zur Darstellung von Knochenstrukturen, während die MRT vor allem zur Beurteilung von Weichteilen eingesetzt wurde. Dies liegt daran, dass Gewebe mit stark gebundenen Wassermolekülen – wie Knochen, Sehnen, Knorpel und Bänder – ein schnell zerfallendes T2*-Signal erzeugen, das von konventionellen MRT-Sequenzen nicht erfasst wird. Um diese Einschränkung zu überwinden, wurden Spoiled-Gradienten-Echo-Sequenzen weiterentwickelt und Sequenzen mit sehr kurzer Echozeit eingeführt, die eine strahlungsfreie Knochendarstellung ermöglichen.

**Methoden:**

Eine gezielte Literaturrecherche in den Datenbanken PubMed und Google Scholar wurde mit den Schlagwörtern „blackbone MRI“, „sCT bone“, „ZTE“, „UTE“, „VIBE“ und „FRACTURE“ durchgeführt. Berücksichtigt wurden englisch- und deutschsprachige Artikel, mit Schwerpunkt auf Studien der letzten fünf Jahre zur Anwendung dieser Sequenzen an Schädel und Wirbelsäule. Zur technischen Einordnung wurden ergänzend ältere Arbeiten herangezogen. Insgesamt ergab die Suche rund 250 relevante Studien zur klinischen Anwendung und 868 zur technischen Grundlage. Nach Auswahlkriterien wurden 69 Studien einbezogen.

**Ergebnisse und Schlussfolgerung:**

Gradientenecho-Techniken wie VIBE, STAR-VIBE und FRACTURE (mit Dixon) verbessern den Knochen-Weichteil-Kontrast. Short-TE-Sequenzen wie ZTE und UTE unterdrücken Weichteilsignale und erlauben eine präzise Knochendarstellung. In Kombination mit KI-Methoden bieten sie langfristig eine strahlungsfreie Alternative zur CT bei traumatischen, neoplastischen und degenerativen Veränderungen.

**Kernaussagen:**

- ZTE ist die bevorzugte Sequenz bei Pathologien des Schädeldachs, präoperativer Bildgebung und für die Erstellung von synthetischem CT (sCT).
- Modifizierte UTE-Sequenzen eignen sich besonders gut für die Bildgebung des Viszerokraniums (DURANDE) und der Wirbelsäule (3D-Stack of Stars UTE mit Dixon-Rekonstruktion).
- VIBE/STAR-VIBE werden für die präoperative und traumatische Gesichtsbildgebung verwendet.
- Quantifizierung der Knochen- und Bandmatrix mit Dual-Echo und IR-Cones-UTE, wobei Parameter wie Porositätsindex, Suppressionsverhältnis und Mapping-Werte ermittelt werden.

## Introduction

Visualization of bone structure in MRI imaging is a challenging task due to its ultra-fast signal decay that cannot be captured by the conventional MRI sequences. Bone water exists in four compartments: free/pore water, water loosely bound at the collagen-mineral interface, water tightly bound within collagen triple helices, and structural water within the mineral. Water within bones plays a critical role in their mechanical properties: free water (~4% of total bone volume) resides in the Haversian and lacuno-canalicular systems, inversely correlating with bone strength and stiffness. Bound water exists in three forms: within collagen triple helices (contributing to toughness), at the collagen-mineral interface (modulating stress and elasticity), and as structural water in apatite crystals (enhancing mineral stability) 1 2 .

Advances in imaging, such as ultrashort echo time (UTE) and zero echo time (ZTE) have enhanced visualization of tightly bound bone water 3 4 5 6 7 . These techniques surpass conventional MRI sequences but still face challenges in spatial resolution and signal-to-noise ratio compared to CT. The motivation of developing new MRI bone techniques was also to avoid the radiation burden from CT, especially in vulnerable populations like children, pregnant women, and individuals requiring frequent imaging. These were intensively explored in the past decade and are extensions of the gradient echo sequence, since this type of sequence guarantees fast imaging that can be flexibly modified by implementation of different gridding techniques, inversion pulses, and suppression pulses ( Table 4 ).

**Table:** **Table 1** MRI gradient echo sequence acronyms.

	**Basic**	**Fast**								
	**Steady-state-longitudinal**	**Steady-state transversal**								
		**RF-Spoiled/ incoherent**				**Refocused-coherent**				
	**Basic**		**Ultrafast gradient echo 2D with preparation pulse**	**Ultrafast gradient echo 3D with preparation pulse**	**Volume-interpolated 3D GRE**	**partially rephased offset average**	**unbalanced/resonant**	**completely rephased (bSSFP)**	**Balanced**	**DESS/FADE**
	**T1**	**T1 **	**T1 **	**T1 **	**T1 **	FID-like post excitation T2*/T1	**Spin Echo-like preexcitation T2**	**2 echoes simultaneously T2/T1**		**FID + Echo** 2 echoes separated T1/T2*, T2
Siemens	GRE	FLASH	TurboFLASH	MPRAGE 3D FGRE	Vibe/Star Vibe	FISP	PSIF	TrueFISP		DESS
GE	GRE	SPGR	FastSPGR	3D Fast SPGR	LAVA	GRASS	SSFP	FIESTA		MENSA
Philips	GRE	T _1_ FFE	TFE	BRAVO 3D TFE	THRIVE	FFE	T _2_ -FFE	b-FFE		

## Techniques

### VIBE

**Fig. 1:**
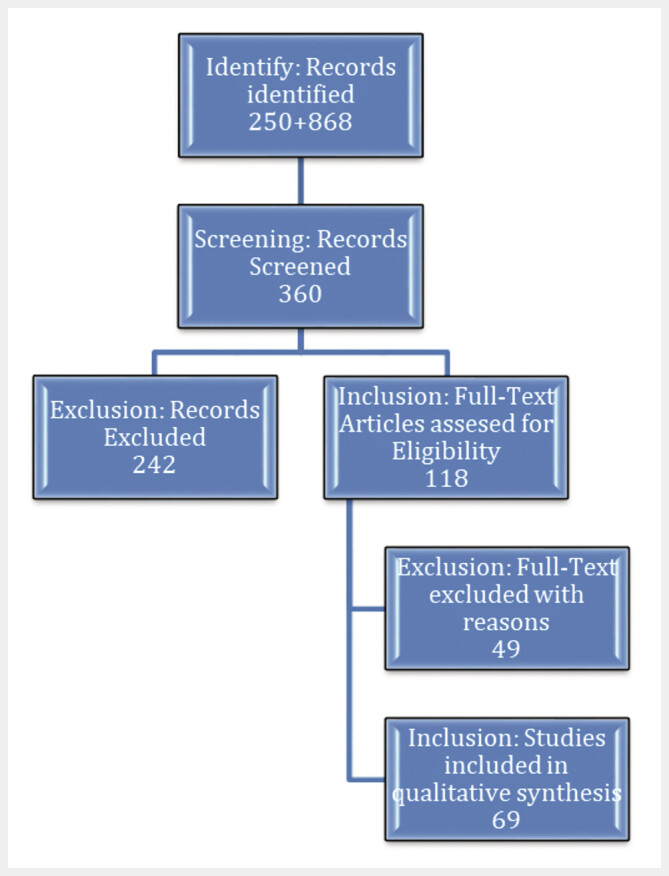
Prisma Flowchart. A total of 250 articles on black bone MRI applications related to the skull and spine were identified, along with 868 articles addressing the underlying technology and physics of the sequences. Based on criteria prioritizing relevance and recency, 360 articles were selected for screening. Of these, 242 were excluded due to either excessive technical detail or a focus on anatomical regions outside the scope of interest. The remaining 118 articles underwent further evaluation, leading to the exclusion of 49 additional studies due to their limited, case-specific applicability.

Modified spoiled gradient echo sequences have been studied extensively over the last decade for their performance in bone imaging. Among them SPGR, Medic, SWI, VIBE/StarVIBE, and FRACTURE show different strengths in MSK imaging ( Table 2 ) ( Fig. 1 ).

**Table:** **Table 2** Characteristics of ZTE, UTE, FRACTURE, VIBE.

Aspect	ZTE (Zero Echo Time)	UTE (Ultrashort Echo Time)	FRACTURE	VIBE (Volumetric Interpolated Breath-Hold Examination)
Relation to SPGR	Derived from gradient echo, with near-zero TE.	Heavily modified gradient echo with ultrashort TE.	Direct derivative of SPGR.	A modified SPGR designed for fast volumetric T1-weighted imaging.
Echo time (TE)	~0 ms	~0.01–0.1 ms	Standard SPGR TE (~1–5 ms).	Very short TE (~1–2 ms).
Repetition time (TR)	Very short (ms range)	Very short (ms range)	Short (5–20 ms).	Short (3–5 ms).
Spoiling	Transverse magnetization is spoiled.	Transverse magnetization is spoiled.	Uses gradient spoiling like SPGR.	Transverse magnetization is spoiled.
Focus	Imaging of tissues with extremely short T2 (bone, teeth).	Imaging of short T2 tissues (tendons, ligaments, cartilage).	High-resolution cortical and trabecular bone imaging.	High-resolution, contrast-enhanced T1 imaging of soft tissues.
Contrast	Proton density–like; highlights short T2 tissues.	Proton density and T2* effects.	T1-weighted with CT-like bone contrast.	T1-weighted contrast, often post-gadolinium.
Signal characteristics	Strong for very short T2 tissues.	Strong for short T2 tissues.	High resolution for cortical bone and trabecular structures.	Optimized for soft tissue and dynamic imaging.
Applications	Cortical bone	Tendons	Occult fractures	Abdominal and pelvic imaging
	Teeth	Ligaments	Bone lesions	Liver and pancreas imaging
	Lung	Cartilage	Trabecular microstructure.	Dynamic contrast-enhanced imaging.
	Orthopedic hardware assessment.	Cortical bone imaging.		
Acquisition speed	Very fast, suitable for 3D imaging.	Fast, suitable for 3D imaging.	Moderate, optimized for high spatial resolution.	Fast, designed for volumetric T1 imaging in breath-hold durations.
Post-processing	Often includes subtraction for bone enhancement.	Sometimes includes subtraction for better contrast.	Includes subtraction for CT-like contrast.	Minimal; typically for dynamic contrast enhancement.
Advantages	Excellent for ultra-short T2 structures.	Captures tissues with short T2 not visible on standard MRI.	High spatial resolution for cortical and trabecular bone.	Rapid acquisition.
	Minimal artifacts.	High sensitivity to cortical bone and tendons.	CT-like imaging for fracture detection.	High-resolution T1 imaging for dynamic contrast-enhanced studies.
	CT-like results without radiation.			
Limitations	Limited soft tissue contrast.	Low specificity for soft tissue contrast.	Not optimal for soft tissue contrast.	Limited utility for bone imaging.
	Susceptibility to noise in short T2 areas.	Susceptibility to artifacts.	Requires complementary sequences for edema detection.	Heavily relies on contrast agents for enhanced results.

The VIBE (volumetric interpolated breath-hold examination) MRI sequence is a 3D gradient echo sequence that uses radio-frequency spoiling to produce T1-weighted three-dimensional images. The main characteristics are breath-holding technique, interpolation, and volumetric imaging. It has improved slice-selective spatial resolution, SNR, and CNR with higher acceleration, thus minimizing motion-related artifacts 8 9 ( Table 3 ).

**Table:** **Table 3** Examples of sequence parameter.

	TR (ms)	TE (ms)	flip angle (°)	FOV (mm)	slice thickness (mm)/voxel size (mm³)*	field strength	scan time (min)	manufacturer	specific
**Skull**									
Petra 10	8.6	4.2	5	240	1	1.5/3		Siemens (Aera, Skyra)	
DURANDE 11	7	0.06/2.40	12	280	/	3	06:00	Siemens (Prisma)	RF width 0.04/0.52 ms
GA-VIBE 12	4.84	2.47	3	192	0.6 × 0.6 × 0.8	3	05:04	Siemens (Prisma, VIDA, Biograph)	azimuthal angle of 111.25°
FRACTURE 13	21	4.61	15	230×230×182	0.7	3	06:48	Philips (Achieva)	echo spacing 4.6 ms
									
**Spine**									
3D stack-of-stars UTE 14	6.3	0.14	5	250×250×279	0.45×0.45×3	3	06:03	Philips (Ingenia)	
VIBE 15	7	2.45		200	2	3		Siemens (Verio)	
ZTE 16	/	0	1	440×440×290	1.6	3	02:50	GE (Signa Premier)	
FRACTURE 17	20.7	4.6	15	230×230×182	0.7	3	07:24	Philips (Achieva)	echo spacing 4.6 ms
									
**sCT with AI**									
3D-T1-MPGR 18	6.5	2.1/3.5/4.8	10	435×435×160	1.2×1.2×2	3	04:38	Philips (Ingenia)	
ZTE 19	5.1	0	2	320	1.5	3	04:06	GE (Discover 750w plus GEM)	
									
**Quantitative UTE**									
3D Dual Echo Cones UTE 20 21	100	0.032/2.2	10	140×140×120	0.87×0.87×5	3	10:00	GE (MR750)	
3D Cones IR-UTE sequence 20 21	100	0.032	20	140×140×120	0.87×0.87×5	3	10:00	GE (MR750)	TI 45 ms
STAIR-UTE-Cones 22	150	0.032	18	300	2.1×2.1×4.5	3	10:00	GE (Pioneer)	TI 64 ms
*Voxel size = Pixel size(x) × Pixel size(y) × Slice thickness									

Interpolation is a crucial tool that enables high-resolution imaging in short acquisition times by estimating and filling in unmeasured data points. It enhances spatial resolution, reduces artifacts, and allows for rapid imaging, making VIBE sequences particularly valuable in abdominal imaging and other applications where quick, high-quality imaging is required. The drawback of partial Fourier interpolation is that if it is not applied carefully, it can introduce artifacts or distortions, particularly in areas with high motion or complex anatomy.

There are different modifications of the sequence that further optimize its performance depending on the anatomical region and the indication. In MSK imaging, fat saturation is usually applied in order to better differentiate cortical bone from fat containing bone marrow and avoid chemical shift artefacts 9 . Innovations of the sequence include the non-Cartesian StarVIBE, which is less prone to motion artifacts, and is particularly useful for preoperative planning 12 23 ( Table 4 ).

**Table:** **Table 4** Comparison of ZTE, UTE, FRACTURE.

Aspect	ZTE	UTE	FRACTURE
Relation to SPGR	Conceptual derivative, but with zero TE.	Modified SPGR with ultrashort TE.	Directly based on SPGR principles.
Echo time (TE)	~0 ms	~0.01–0.1 ms	Standard SPGR TE (~1–5 ms).
Focus	Tissues with very short T2 (e.g., bone).	Tissues with short T2 (e.g., tendons, ligaments).	High-resolution bone imaging.
Applications	Cortical bone, teeth, lung imaging.	Tendons, ligaments, cartilage.	Fractures, bone lesions, microstructure.

### FRACTURE

The FRACTURE MRI, or 3D-multislice FFE sequence, introduced in 2020, is a modified spoiled-echo sequence that delivers results comparable to CT for certain applications ( Table 2 ). It employs a 3D multi-echo gradient echo sequence with in-phase echo spacing to enhance bone contrast, utilizing four echoes with a spacing of 4.6 ms ( Table 3 ). In post-processing, the last echo is subtracted from the sum of all echoes, and the contrast is inverted to simulate CT imaging 17 24 25 26 . This multiecho approach allows the sequence to visualize both short and long T2* tissues simultaneously. Early echoes capture bone signals, while later echoes highlight soft tissue, resulting in high contrast. The sequence features a fast repetition time (TR) of 21 ms and a small flip angle of 15° to maintain a high signal-to-noise ratio. Its isotropic voxel size (0.7 × 0.7 × 0.7 mm) ensures high spatial resolution, enabling detailed imaging and multiplanar reconstructions. The sequence offers a mix of T1 and T2 weighting, making it effective for detecting subtle fractures and bone discontinuities, though it is less sensitive than STIR or T2-weighted sequences for identifying bone marrow edema. Fat-suppression techniques are often incorporated to improve contrast between cortical bone, cancellous marrow, and soft tissue. However, being gradient-echo-based, it is more susceptible to magnetic field inhomogeneity artifacts, particularly near metal implants.

## Non-Cartesian

The choice of sampling grid significantly influences image acquisition, reconstruction, and the types of artifacts present in the resulting images. Cartesian sampling, commonly used in routine imaging, is preferred for its simplicity and speed. In contrast, non-Cartesian sampling is utilized for advanced applications such as imaging short T2 tissues (tendons, bones), motion-prone scenarios (cardiac imaging), and achieving isotropic resolution or efficient 3D k-space coverage. Non-Cartesian techniques sample k-space along trajectories that deviate from the Cartesian (x, y) grid, using patterns like radial, spiral, or petal paths. The sampling often starts at the center of k-space and acquires data in a center-out fashion, oversampling the central region. This makes it robust to motion but also increases acquisition time compared to Cartesian techniques. To address this, undersampling methods like parallel imaging and compressed sensing are applied. Several non-Cartesian trajectories are widely used: radial, spiral, cones, and petal (rosette).

- Radial imaging (projection reconstruction): Acquires data as projections at multiple angles, which are backprojected to form the image. This method offers robustness against motion artifacts.
- Spiral imaging: Follows an Archimedean spiral path in k-space, covering it efficiently with minimal repetition. Spiral acquisitions often outperform radial imaging, delivering better contrast-to-noise ratio (CNR) and spatial resolution with shorter echo times.
- 3D UTE-Cones: Combines short rectangular RF pulses with 3D spiral cone trajectories. Spiral arms are rotated around the k-space z-axis to generate ~10,000 – 40,000 spokes, achieving comprehensive 3D k-space coverage. Applications include T2* and T1 mapping and magnetization transfer quantification for macromolecular fraction measurements 27 .
- 3D UTE-Petal: Utilizes dual echo acquisition with radial and angular sampling, starting and ending each TR at the center of k-space. Crusher gradients in three directions reduce artifacts, while the outer k-space is more densely sampled, enabling higher undersampling factors and improved SNR. This sequence is particularly effective for imaging myelin-rich white matter and cartilage degeneration quantification 28 .
- ZTE: Employs a center-out radial trajectory without slice selection, offering an alternative for imaging short T2 tissues.

The above-mentioned sequences are usually combined with acceleration and reconstruction techniques like parallel imaging, compressed sensing and deep learning-based methods for further optimization 29 30 31 . The deep learning approach can be interpreted as a more advanced version of compressed sensing; it augments parallel imaging by addressing its limitations, such as SNR loss and the impact of the g-factor. Advanced algorithms like Deep Resolve Gain and Deep Resolve Sharp restore SNR and improve image sharpness. Furthermore, AI is not only a tool for image reconstruction and augmentation but also for synthetization, like synthetic CT from MRI, synthetic MRI sequences from other MRI data, synthetic MRI from ultrasound. These techniques are frequently used in combination with non-Cartesian black bone sequences. In the following, we will present the basic characteristics of the two most frequently used black bone sequences in the musculoskeletal imaging-ZTE and UTE.

### ZTE

**Fig. 2:**
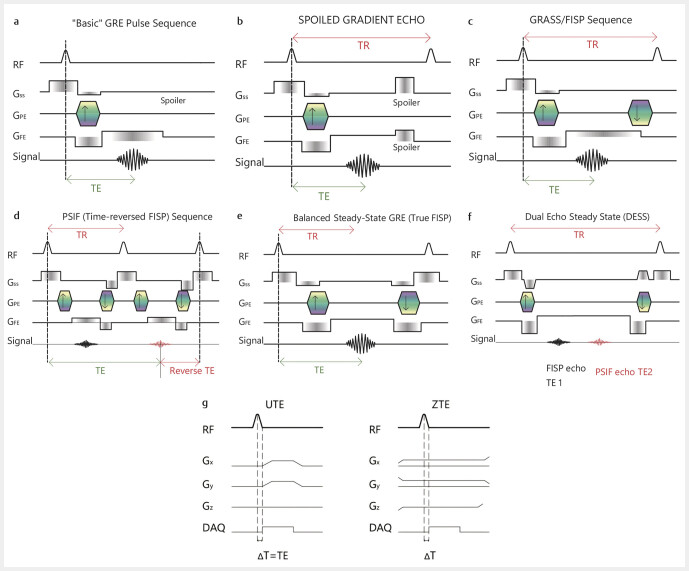
GRE-sequence scheme. **a** Basic – not usually used in their simplest form due to long TR and TE. **b** Spoiled – utilizes RF spoiling to achieve an incoherent steady state by eliminating residual transverse magnetization through phase dispersion, ensuring consistency in T1-weighted imaging. **c** GRASS/FISP – unbalanced refocused sequence, uses gradient spoiling with constant gradients, leading to the averaging of resonant offset frequencies. Reads out the free induction decay (FID) signal, more T1-weighting. **d** PSIF – inverted GRASS/FISP, reads the echo signal that comes after the second RF pulse, which contains more T2-like contrast, which is sensitive to fluids, however it is largely replaced by the balanced GRE-sequence. **e** Balanced – balanced refocused sequences implement phase cycling and precise refocusing gradients along the phase-encoding direction to achieve full gradient balancing. This ensures optimal preservation of phase coherence and minimizes signal loss due to dephasing. They refocus the FID and echo-like signal in a single echo, which enhances T2/T1 contrast and highlights fluid. **f** DESS – similar to the balanced GRE collects FID and echo-like signal, however the two signals are processed separately to extract different tissue properties, mixed T1/T2, used mainly for cartilage imaging. **g** ZTE + UTE – use very short TE with fast mode switching of the coils from transmitting to receiving, can directly capture bone signal.

Zero echo-time (ZTE) MRI is a novel imaging technique that utilizes ultrafast image acquisition immediately after applying the radiofrequency pulse resulting in near-zero echo times ( Table 2 ) ( Fig. 2 ). After initial data readout gradient spoiling, adjustment and settling are rapidly performed, followed by the next radiofrequency pulse with very short repetition times, thus making it fast, silent and artifact resistant 4 7 32 ( Table 3 ).

The precursor of the ZTE is described in 1995 by David P. Madio as RUFIS (rotating ultra-fast imaging sequence) that uses FID (free induction decay) instead of echoes, which makes it insensitive to motion, flow and diffusion 7 33 . At a later stage, the first black bone sequence was described by Eley in 2012 in craniofacial imaging 33 . She uses a repetition time of 8.6 ms and an echo time of 4.2 ms, with a flip angle of 5° for optimal suppression of fat and water.

ZTE acquires free induction decay (FID) signal that does not rely on gradient echo or spin echo signal refocusing and starts immediately after excitation, thus the RF excitation is entangled with the image encoding readout gradient, which precludes slice selection. However, the MR system can start sampling the FID signal only after RF hardware changes state from transmit to receive. This gap time depends on the RF coil and system characteristics and must be under 24 μs, some use 8 μs. The sampling of the signal for the outer portion of k-space occurs in a non-Cartesian scheme, along radial trajectories, which enables flexibility and efficiency of k-space sampling, motion insensitivity, and image generation with high spatial-temporal resolution from limited data.

Because of the finite transmit-receive switching delay, the first few data samples of the center-out 3D radial spokes are missing resulting in a spherical gap of samples at the center of k-space and data is acquired starting at some minimum k-space radius. There are three ways to fill the center k-space gap using PETRA (pointwise encoding time reduction with radial acquisition), WASPI (water- and fat-suppressed solid-state proton projection imaging), and HYFI (hybrid filling) 32 .

Analogous to multiecho GRE or UTE, ZTE can be turned into a multi echo sequence that generates positive bone contrast by subtracting a later in-phase gradient-echo from the TE = 0 FID image, using the looping star sequence multiple FID signals are first excited and then sequentially gradient refocused in a looping, time multiplexed manner 7 .

### UTE

The UTE and ZTE techniques were developed to directly visualize short T2* tissues. In order to capture the fast-decaying signal from bones, they use very short and zero TE ( Table 2 ) ( Fig. 2 ). In contrast to ZTE, data acquisition doesn't begin during RF excitation in UTE, thus the TE is not zero 5 6 ( Table 3 ). A half-pulse excitation is commonly used in the 2D sequence, where two acquisitions with alternating slice select gradients are summed to obtain the full-pulse slice profile. The more efficient 3D acquisition does not require slice selective excitation and instead uses a hard pulse, which eliminates several problems like sensitivity to timing errors, eddy current artifacts, etc. The downside of the 3D acquisition is the longer scan time and shimming over a large volume.

UTE is used not only for qualitative but also for quantitative evaluation of the microstructure of bones, cartilage, and tendons. Many studies have shown that the bone mineral density (BMD) measurement has low correlation with bone strength and a poor fracture prediction rate of 30–50% 20 21 34 35 . Therefore, more sensitive assessment tools have been created, evaluating bone microstructure, porosity, organic matrix, bone water, and bone perfusion.

The bone porosity can be measured with micro-computed tomography (μCT) with high spatial resolution of 82 μm, an alternative method is the UTE sequence, including dual-echo techniques, echo subtraction, inversion recovery, spectroscopy, and phase imaging which are explained in Table 5 5 36 .

**Table:** **Table 5** UTE technique types.

Sequence name	Technique	Pros	Cons
**Dual-Echo UTE with Echo Subtraction (dUTE)**	Captures signals from both short- and long-T2 components in the first echo; subtraction isolates short-T2 signals.	Simple, time-efficient, provides high short-T2 contrast.	Noise, eddy currents, susceptibility effects, residual long-T2 signals if echo spacing is too long.
**Dual-Echo UTE with Rescaled Subtraction (UTE-RS)**	Scales down the FID signal in the first echo to suppress fat and muscle signals during subtraction.	Enhanced contrast for short-T2 species like cortical bone, minimizes soft tissue artifacts.	Requires accurate scaling to prevent residual artifacts.
**Long-T2 Saturation UTE (sUTE)**	Applies a 90° saturation pulse followed by a crusher gradient to suppress long-T2 magnetization; short-T2 signals are minimally affected.	Effective suppression of long-T2 water and fat.	Residual long-T2 signals from short-T1 species like fat; requires subtraction for cleaner imaging.
**UTE with Off-Resonance Saturation (UTE-OSC)**	Uses off-resonance RF pulses for selective suppression of bound water and collagen protons through direct saturation, cross-relaxation, or exchange.	High contrast for cortical bone.	Partial suppression of periosteum; susceptibility artifacts near bone-periosteum interfaces.
**Single Adiabatic Inversion Recovery UTE (SIR-UTE)**	Uses an adiabatic inversion pulse to null long-T2 components (water and fat).	High SNR and CNR between bone and soft tissues.	Residual long-T2 signals require subtraction.
**Dual Adiabatic Inversion Recovery UTE (DIR-UTE)**	Successively inverts water and fat signals with different inversion times (TI1 and TI2) for simultaneous suppression.	High robustness, uniform suppression, excellent cortical bone contrast.	Increased acquisition complexity and time.
**Ultrashort-TE Spectroscopic Imaging (UTESI)**	Combines UTE with undersampled interleaved multi-echo acquisitions; uses chemical shifts and resonance frequency shifts for spectral decomposition.	Provides information on chemical composition, bulk magnetic susceptibility, and phase evolution.	Lower spatial resolution compared to standard UTE techniques.
**UTE Phase Mapping**	Uses phase shifts caused by susceptibility effects in cortical bone for high-contrast imaging.	Highlights susceptibility-related properties, useful for distinguishing water in bone’s Haversian system.	Susceptible to phase distortions from field inhomogeneity.
**Direct Imaging of Bound and Free Bone Water**	Differentiates water bound to organic matrix (short T2*) and free water in pores (longer T2*).	Provides critical data on bone porosity and matrix density.	Requires high SNR, precise signal modeling, and external water phantoms for quantification.
**Magnetization Transfer (MT)**	Applies off-resonance saturation to bound water, allowing exchange with free water for quantification.	Enables evaluation of bound/free water interactions, reveals insights into organic matrix integrity.	Conventional MT methods are limited to long-T2 tissues; UTE adaptations required for cortical bone.
**Bone Perfusion Imaging**	Uses dynamic contrast-enhanced UTE imaging to track blood flow in cortical bone.	Correlates with bone remodeling and metabolic activity; useful for assessing vascularity in conditions like osteoporosis or post-surgery.	

These techniques are used for better depiction of the short-decaying signal of bones and suppression of the long-T2 tissues. The high spatial resolution, SNR, and CNR of these sequences allow the measurement of T1 and T2* value, the assessment of perfusion, the distinction of cortical bone from periosteum, the quantification of bone water content, the visualization of fracture healing, the measurement of phosphorus and sodium content and the evaluation of the magnetization transfer ratio (MTR). These applications are illustrated in Table 7 .

**Table:** **Table 6** UTE mapping technique.

Name	Principle	Target	Data processing
T2*-mapping	T2* mapping quantifies the transverse relaxation time affected by magnetic field inhomogeneities and local susceptibility	water content	Step 1: Image acquisition with 3D UTE at different echo times (e.g. 50 µs, 0.1 ms, 0.2 ms). Step 2: Image registration, masking Step 3. Fitting voxel-wise with Log-linear, nonlinear (Levenberg-Marquardt) or hybrid (ARLO) Step 4: Generate T2* map – store the fitted T2* value for each voxel in a map. Optional smoothing or masking.
T1-mapping	T1 mapping involves acquiring images at different time points or inversion recovery times to generate a voxel-wise map(or ROI-based) of the T1 relaxation time, which reflects how quickly protons realign with the magnetic field after excitation. There are different mapping methods using specific mapping functions-Saturation recovery, inversion recovery, VTR/STR (variable or single TR), AFI/VFI(actual or variable Flip angle).	cortical bone, to assess cortical bone porosity, monitoring temperature change, for other quantitative methods like MT	Step 1: Data acquisition (using UTE) at different TRs (Variable TR method) or at different flip angles (Variable Flip Angle), Step 2: Signal model – fit the measured S(TR) to the exponential curve to estimate T1 Step 3: Curve fitting (Nonlinear Least Squares)-typically done using Levenberg-Marquardt algorithm (standard for nonlinear least squares), or gradient-based optimizers (Adam in machine learning contexts). curve_fit (Python), lsqcurvefit (MATLAB) Step 4: Output – after fitting, for each voxel you get T1 value: used to create a T1 map
T1rho-mapping	T1ρ (spin-lattice relaxation in the rotating frame) measures how spins relax while being “locked” in the transverse plane with a spin-lock RF pulse, which is applied parallel to the tipped magnetization vector after the initial 90° pulse. The decay of this locked magnetization to 0 is the T1 ρ time.	Proteoglycan, glycosaminoglycan, sensitive to slow molecular motion, macromolecular interactions and early degenerative changes in tissues.	Step 1: Spin-lock preparation: spin-lock pulse of strength B1 for varying durations TSL (Time of Spin Lock). Step 2: UTE readout Step 3: Acquire multiple TSLs-acquire a series of images at different TSL values Step 4: Model the signal Step 5: Fit the curve – with nonlinear least squares (Levenberg-Marquardt), log-linear, or ARLO to fit the T1ρ value for each voxel or ROI
MT (magnetization transfer)	Indirect estimation of the macromolecule content by first saturation of the bound water-signal with an off-resonance saturation pulse, after interaction with the free water (due to cross-relaxation or chemical exchange), the saturation is partially passed to the latter, which can be measured ,UTE-MT technique provides indirect information about collagen content and integrity by utilizing the exchange effect.	collagen structural integrity	Step 1: Two sets of UTE image acquisition with MT pulse (off-resonance RF saturation) and without MT pulse (control) Step 2: Rigid and non-rigid registration Step 3 (optional): Signal normalization like B1/B0 corrections (preprocessing) Step 4: MMF calculation by fitting the data using the modified two-pool MT model Step 5: MTR calculation – signal difference between the MT data with almost no MT effect and the data with maximum MT effect normalized to the signal of the data with almost no MT effect.

**Table:** **Table 7** Applications of the UTE techniques.

Target structure	Anatomical region	Diagnosis	Pathology	Signal	Sequence of choice/method
tendon	knee, shoulder, Achilles	degradation	Stage 1 PG und cross-links increase Stage 2 degeneration, microtear, degradation leading to collagen distortion → water permeability → decreased collagen bound water and increased free water.	Stage 1: T2 increase, T1 decrease, MTR decrease Stage 2: T2 increase, T1 increase , MTR decrease	UTE MT outperforms T2* and T1 mapping in differentiating partial tendon tears.
	knee	graft healing after ACL reconstruction	Central graft necrosis and hypocellularity, proliferation-phase, ligamentization-phase	UTE-T2* value increase at 6 months, decrease between 6 and 12 months after operation UTE-T2* and Tρ-increase with inferior graft properties	3D Cones-UTE-T2*- and T1rho-mapping, in a sagittal-oblique plane
cartilage	knee, hip	osteoarthritis	Mankin-histology classification-surface irregularities, decreased cellularity, increased cell cloning, tidemark integrity loss	UTE-T2*-increase, correlated with water contents, UTE-MTR-decrease, correlated with collagen, UTE-AdiabT1ρ decrease, correlated with GAG and collagen.	UTE-MTR and UTE-AdiabT1ρ better depict early degeneration than UTE-T2* and T2 and T1ρ . Recommended sequence: 3D UTE with AdiabT1ρ, 3D Cube Quant-T1ρ.
cartilage overload	knee	cartilage overload	Mild degenerative damage induced by fatigue loading, reversible.	UTE-MTR decrease after 2 days, increase after 4 weeks UTE-T2* increase after 2 days, decrease after 4 weeks	UTE MT better than UTE-T2* in detecting dynamic changes before and after sports activity
	knee	DM/PAD (peripheral arterial disease)	Increased mineralization of the deep cartilage layer, short-term functional adaptation to protect the hyaline cartilage, associated with micro- and macrovascular disease. Cartilage loss in the long term.	UTE-T2* decrease in deep cartilage	3D Cones-UTE-T2*-mapping
disc	spine	osteochondrosis	Pfirrman classification: 1. Early stage: reduction of PG content and dehydration, increase in collagen II of annulus fibrosus. 2. Advanced stage: reduction in disc height, decrease in annulus fibrosus/nucleus pulposus differentiation. The disc becomes friable and fissures and degradation occur.	UTE-T2* decrease with increasing degree of degeneration T1ρ value decrease, both negative correlation with Pfirrmann grade	UTE-T2* and UTE-Adiab-T1ρ-mapping , UTE-mapping (biochemical information on the disc ultrastructure) better than standard T2*-mapping (hydration), UTE catches the short T2* relaxations from the intervertebral tissue,
endplate	spine	osteochondrosis	Modic classification: inflammation, yellow fatty marrow-formation, sclerosis	Healthy: bright continue line of the end plate; degeneration: discontinuity or irregularity.	3D IR-FS-UTE (real signal detection of the end plate) better than Dual-echo UTE (low CNR, more artifacts), method: UTE-VFA-T1-mapping
bone	whole body	osteoporosis	reduction in bone mass and microarchitectural deterioration, thin and disconnected trabeculae, increased osteoclastic activity, thinning of cortical bone	CBWPD, BMD, and T score decrease, BMFF-increase in osteoporosis PI increase, SR-increase in osteoporosis	IDEAL-IQ for BMFF-quantification, 3D STAIR-UTE Cones for CBW imaging( suppression of the trabecular bone-signal), Dual-Echo-UTE for porosity index, IR-UTE for suppression ratio

### sCT

Synthetic CT or bone MRI can be acquisition-based or reconstruction-based, the former using a dedicated bone-sensitive sequence like ZTE/UTE and the latter using DL reconstruction-methods, mainly from 3D T1 multi-phase gradient echo sequences 16 . Synthetic CT is generated for MRI-only radiotherapy planning, surgery-planning, radiation-free diagnostic imaging and MRI-only workflows 37 38 39 40 .

Deep learning techniques for synthetic CT-generation include CNN and GAN methods, the former is generator-based and the latter relies on both a generator and a discriminator. Compared to generator-only models, GAN introduces a data-driven regularizer — the adversarial loss — to ensure that the learned distribution approaches the ground truth. This prevents the generated images from blurring and better preserves details, especially edge features; the accuracy of sCT within the bone region is increased; and the discriminator detects patch features in both real and fake images mitigating misregistration problems caused by an imperfect alignment between multi-parametric MRI and CT. This is why some studies state that cGAN is better in sCT generation than CNN only. U-Net and DUNet are useful for simpler applications or where stability and anatomical consistency are prioritized over fine-grained image realism. However, for high-quality synthetic CT generation, GANs (especially Pix2Pix or CycleGAN) are generally preferred due to their ability to produce sharper, more realistic images. The drawback of the GAN-network is that training can be unstable.

As stated above the main reason for MRI-to-CT translation is the field of radiation therapy, but recent advances have shown good results of synthetic CT for diagnostic purposes and surgery planning. With the aid of deep learning sufficient resolution and image quality could be achieved for detecting fractures and tumors and diverse quantification methods are being augmented for measuring bone density and for preoperative planning.

The state-of-the-art MRI sequence for generating sCT is the 3D T1 multi-echo spoiled gradient echo sequence, but some studies showed that using a dedicated bone-MRI sequence is more beneficial 41 ( Table 3 ). Other than that, especially in the spine, spin echo sequences have been successfully used. Another consideration is how many sequences can be used for input. In studies, multiple sequences proved better especially in converging the MR signal into HU, because more sequences yield more information for the differentiation between different tissue types. The following chapter will explore the use of the above-mentioned techniques in the skull and spine region.

## Skull

**Fig. 3:**
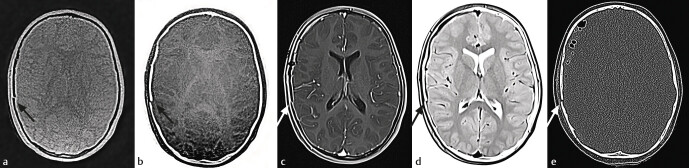
Comparison of ZTE vs. VIBE vs. CT for detection of skull fracture. **a** -zte normal – well delineation of the skull fracture, failed visualization of the pneumocranium. **b** -zte-inverted. **c** -vibe normal – captures additional findings like epidural hematoma. **d** -vibe-inverted. **e** -CT – yields the best quality in fracture depiction and intracranial air detection.

**Fig. 4:**
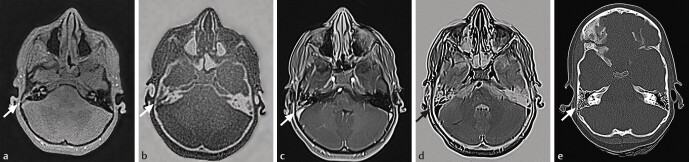
Comparison of ZTE vs. VIBE vs. CT for detection of mastoid fracture. **a** -ZTE normal – depicts the fracture line better than VIBE or the inverted ZTE, however with lower resolution compared to CT. **b** -ZTE-inverted – worse depiction of the fracture due to inversion of the usual signal drop-out of the air, constrained air/bone differentiation. **c** -VIBE normal – depicts more of the soft tissue. **d** -VIBE-inverted. **e** -CT.

In the skull region, black bone sequences can be performed for traumatic, inflammatory, neoplastic, and developmental conditions with a nearly comparable image quality to CT 10 11 12 13 33 42 43 44 ( Table 8 ) ( Fig. 3 , Fig. 4 ).

**Table:** **Table 8** Comparison of MRI sequence for visualizing short T2* tissue.

Anatomical region	Authors and year	patient population	indication	Sequence	Other sequences	Quality metric	Results	Limitation
**Skull**								
	Zero TE MRI for Craniofacial Bone Imaging, A. Lu, 2019 45	in vivo, children, case series	fracture, anomaly, postoperative, neoplasia	ZTE	CT	qualitative	comparable to CT	physician awareness, scanner and hardware compatibility, vendor sequence availability, clinical workflow, image post processing and analysis.
	Does the Addition of a “Black Bone” Sequence to a Fast Multisequence Trauma MR Protocol Allow MRI to Replace CT after Traumatic Brain Injury in Children? X M.H.G. Dremmen, 2017 10	in vivo, children with head trauma	fracture, hemorrhage	ZTE	3D-T1	sensitivity, specificity, PPV, NPV	66–87% vs 100% (CT)	bone/air-interface, non-displaced linear fractures
	Evaluation of ultrashort echo-time (UTE) and fast-field-echo (FRACTURE) sequences for skull bone visualization and fracture detection − A postmortem study, Eva Deininger-Czermaka, 2021 44	ex vivo, 20 subjects	fracture detection	FRACTURE/3D-FFE-inphase, 2D-UTE	CT	ICC, Lickert, SNR, CNR	ICC: 0.75, Lickert: 2.6–2.8, CNR-CT>UTE>FRACTURE, SNR-FFE>UTE	pneumatized spaces, chemical shift, inhomogeneity of the magnetic field
	FRACTURE MRI: Optimized 3D multi-echo in-phase sequence for bone damage assessment in craniocerebral gunshot injuries, D. Gascho, 2020 13	ex vivo, 4 subjects	craniocerebral gunshot wounds, fracture-detection	FRACTURE/3D-FFE-inphase	3D-T1, 3D-T2, CT	Likert-scale	equivalent to CT, higher score than T2- and T1-weighted	small bone fragments and gas
	3D pediatric cranial bone imaging using high-resolution MRI for visualizing cranial sutures: a pilot study, Kamlesh B. Patel, 2021 12	in vivo, 11 patients	craniosynostosis	3D-GA-VIBE(STAR-VIBE)	CT	sensitivity, specificity, ICC	97% and 96%, ICC-1.00	manual selection of the appropriate signal threshold for bone separation
	Cranial bone imaging using ultrashort echo-time bone-selective MRI as an alternative to gradient-echo based “black-bone” techniques, Nada Kamona, 2024 11	in vivo, 10 healthy subjects	anatomical delineation, mask generation with histogram-based approach	DURANDE	ZTE-PETRA, GRE	Dice coefficient	81%(DURANDE vs ZTE)	blurring caused by off-resonant, not manufacturer supported, post-processing(bias field correction, logarithmic inversion, gradient delay calibration
**Spine**								
	Zero Echo Time Musculoskeletal MRI: Technique, Optimization, Applications, and Pitfalls, Üstün Aydıngöz, MD, 2022 46	case series	Spondylolysis and spondylolisthesis, spondylodiscitis and osteomyelitis	3D-ZTE	CT, conventional MRI	visual scala	pars articularis fracture better than conventional	tendons and ligaments are not completely suppressed, intra articular gas and hemosiderin
	VIBE MRI: an alternative to CT in the imaging of sports-related osseous pathology?, Eamon Koh, 2018 47	young athletes	pars fracture	3D-VIBE	CT	accuracy, sensitivity, specificity	100, 96, 92	incomplete pars fractures
	Magnetic resonance bone imaging: applications to vertebral lesions, Kazuhiro Tsuchiya, 2023 48 49	in vivo, healthy subjects and patients	degenerative diseases, tumors and similar diseases, fractures, infectious diseases, and hemangioma.	3D-VIBE	ZTE/UTE, SWI, CT		UTE/ZTE: cortical bone abnormalities, 3D VIBE: both bone and soft tissue	
	CT-like images based on T1 spoiled gradient-echo and ultra-short echo time MRI sequences for the assessment of vertebral fractures and degenerative bone changes of the spine, Benedikt J. Schwaiger, 2021 50	in vivo, 30 patients	anterior/posterior vertebral height, fracture age; disc height, neuroforaminal diameter, grades of spondylolisthesis, osteophytes, sclerosis, and facet joint degeneration.	T1SGRE and UTE.	CT	accuracy and agreement, ICC, Lickert	T1–95%–97%, UTE: 91%–95% ,ICC-0.99	metal artifacts, no severe fracture patterns, pathologic fractures or bone metastases.
	Diagnostic value of water-fat-separated images and CT-like susceptibility-weighted images extracted from a single ultrashort echo time sequence for the evaluation of vertebral fractures and degenerative changes of the spine, Georg C. Feuerriegel, 2022 14	30 patients	acute vertebral fractures	sUTE-Dixon	STIR, T1	ICC, Lickert	0.90, almost perfect agreement for the classification and detection of vertebral fractures	fat blurring, venous plexus mimics fracture, susceptibility and chemical shift effects
**OP**								
	A novel black bone MRI protocol for optimization of 3D head and neck resection planning, Hoving, A.M., 2016 51	3 volunteers (test), 10 patients (validation), 2 surgeries (clinical value)	virtual 3D/MRI based surgical planning for mandibular resection, exclusion of the multimodality component in preoperative workflow	3 black bone MRI-VIBE, VIBE + fat sat + GRAPPA, VIBE out-of-phase + GRAPPA	CT	surface deviation analysis, postoperative	mean deviation values: 0.56, 0.50 and 0.58 mm for the three black bone MRI sequences. The most adequate segmented sequence was black bone with FAT SAT + GRAPPA	poor segmentation quality in the mental region with fat sat, poor segmentation quality of the mandibular angles in out-of-phase, metal dental implants-image distortion especially with fat sat, GRAPPA leads to increased noise, coronoid process and the mandibular condyles are difficult to separate from muscle attachment
	High resolution MR for quantitative assessment of inferior alveolar nerve impairment in course of mandible fractures: an imaging feasibility study Egon Burian, 2020 52	15 patients with unilateral mandible fractures involving the inferior alveolar nerve	assessment of both mandible fractures and IAN damage, risk of permanent hypoesthesia, risk of IAN injury in wisdom tooth removal, implant placement and orthognathic surgery	3D STIR, 3D DESS and 3D T1 FFE	CT	Apparent nerve-muscle contrast-to-noise ratio (aNMCNR), apparent signal-to-noise ratio (aSNR), nerve diameter and fracture dislocation	significant increase of aNMCNR, aSNR and nerve diameter in nerve injury, T1 FFE dislocation depiction comparable to CT, DESS-reliable depiction of nerve topography.	anatomical peculiarities like atrophied mandibles must be investigated, implants, metallic restorations or osteosynthesis material-related artifacts may reduce the image quality.
	Robot-Assisted Lumbar Pedicle Screw Placement Based on 3D Magnetic Resonance Imaging Franziska C. S. Altorfer, MD, 2024 53	ex vivo, human cadaver	accuracy of MRI-guided robotic-assisted pedicle screw placement in the lumbar region	ZTE, SPGR	CT	median deviation, pedicle breach (safe <2mm)	median deviation: 0.25 mm, in the axial plane 0.27 mm, in the sagittal plane 0.24 mm, pedicle breach-1,3mm, SPGR has higher SNR, lower CNR (ligament/cortical bone), ZTE less motion artifacts, MRI signal converted into HU.	limited sample size, ex-vivo: not normal surgical circumstances, no respiration-associated artifacts, only one region of the spine, poor generalization
**UTE**								
	Ultrashort Echo Time (UTE) MRI porosity index (PI) and suppression ratio (SR) correlate with the cortical bone microstructural and mechanical properties: Ex vivo study Saeed Jerban, 2023 20	ex vivo, 37 cortical bone strips from tibial and femoral mid shafts	correlation between PI/SR and microstructural and mechanical bone properties	dual-echo 3D-Cones UTE (porosity index PI), IR-3D-Cones-UTE (suppression ratio SR)	μCT	average bone mineral density, porosity, and pore size, 4-point bending test	significant correlations with PI (R=0.68–0.71) and moderate with SR (R=0.58–0.68), stress tests: moderate correlations with PI and SR (R=0.52–0.62), bone mechanical properties: lower for specimens with higher PI and SR.	in vivo: reduced performance due to fat, muscles, and other soft tissues, higher body temperature, subject motion, PI correlated better with microstructural and mechanical parameters than SR, SR depends on the selection of TR and TI, IR-UTE (shorter TR/TI: better pore water-suppression)
	MRI-based porosity index (PI) and suppression ratio (SR) in the tibial cortex show significant differences between normal, osteopenic, and osteoporotic female subjects Saeed Jerban, 2022 21	in vivo, female, 37 (normal), 14 (osteopenia (OPe)), 31 (osteoporosis (OPo))	PI, SR, bone thickness	dual-echo 3D-Cones UTE (porosity index PI), IR-3D-Cones-UTE (suppression ratio SR)	(DEXA) T-score	PI, SR, R	PI (OPo) > PI(normal-24% and OPe-16%), SR (OPo) > SR (normal-41% and OPe-21%), SR (OPe)> SR (normal-16%), Cortical bone (OPo) < Normal (22%) and OPe (13%), DEXA T: correlates poor with PI (R=-0.32), moderate with SR (R=-0.50), and moderate with bone thickness (R=0.51).	further correlation with HR-pQCT or DEXA needed, tibial bone: not the most prominent fracture site in OPo, UTE of the hip or spine must be performed, however due to the thinner bone with sophisticated morphology and deeper localization – more difficult
	Assessment of Osteoporosis in Lumbar Spine: In Vivo Quantitative MR Imaging of Collagen Bound Water in Trabecular Bone Jin Liu, 2022 22	189 participants, mean age-56 y, lumbar spine, normal, OPe, OPo	Fracture Risk Assessment Tool (FRAX). Lumbar CBWPD, bone marrow fat fraction (BMFF), bone mineral density (BMD) and T score	3D short repetition time adiabatic inversion recovery prepared ultrashort echo time (STAIR-UTE)	qCT, DXA	CBWPD, BMFF, BMD, FRAX, T score, R	CBWPD-strong correlation with BMD (R2 = 0.75) and T score (R2 = 0.59), moderate correlation with FRAX score (R2 = 0.48), CBWPD differentiates well between the three different subject cohorts, CBWPD has better correlations with BMD, T score, and FRAX score than BMFF, BMFF does not reveal true bone loss: normal BMFF-but still bone loss or abnormal bone mineralization,	long scan time-12 min (CS and PI can help), no follow-up of the fracture rate,
	Comprehensive assessment of in vivo lumbar spine intervertebral discs using a 3D adiabatic T1ρ prepared ultrashort echo time (UTE-Adiab-T1ρ) pulse sequence Zhao Wei, 2022 54	in vivo, 17 subjects, lumbar spine-segmented into seven regions (outer anterior annulus fibrosus, inner anterior annulus fibrosus, outer posterior annulus fibrosus, inner posterior annulus fibrosus, superior CEP, inferior CEP, and NP).	T1ρ of cartilaginous endplates (CEPs), intervertebral discs (IVDs), nucleus pulposus (NP), CEP may contain both short and long T2 water components.	UTE-Adiab-T1ρ	T2-FSE, Pfirrmann grades	R	T1ρ values of the outer posterior annulus fibrosis, superior CEP, inferior CEP, and NP-moderate correlation with modified Pfirrmann grades with-R : 0.51, 0.36, 0.38, and −0.94, moderate correlations of T1ρ values of the outer anterior annulus fibrosus, outer posterior annulus fibrosis, and NP with age-R: 0.52, 0.71, and −0.76, significant T1ρ differences of the outer posterior annulus fibrosis, inferior CEP, and NP between the subjects with and without lumbar back pain, NP-inverse due to loss of proteoglycans during the process of degeneration	long train of spokes can introduce signal variation along the spokes in a single TR, which may affect the image quality, lower image resolution compared to the thickness of the CEP, trade-off between spatial resolution, scan time, SNR: advanced RF coil with higher SNR can allow improvement of spatial resolution without compromising scan time.

**Fig. 5:**
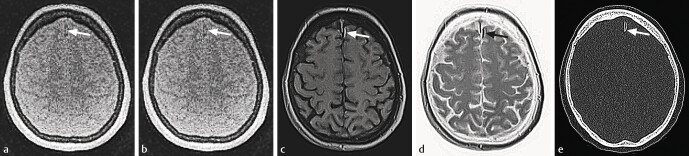
Comparison of ZTE vs. VIBE vs. CT for detection of calcification. **a** -zte normal – better depicts the calcification of the falx compared to vibe, but lacks spatial resolution. **b** -zte-inverted. **c** -vibe normal – produces paradoxical high signal in the area of calcification with respectively false signal drop-out when inverted. **d** -vibe-inverted. **e** -CT.

With regard to anatomical delineation ZTE achieves a high degree of accuracy, which enables accurate segmentation for operative planning or radiation therapy planning, with only 0.32 mm average discrepancy to CT, according to Eley 33 . In studies of healthy subjects, ZTE surpasses FRACTURE and UTE in imaging the cranial vault, but in the skull-base and viscerocranium novel UTE-approaches hold the potential to outperform the rest, thanks to their multiecho approach with subtraction 4 13 33 42 43 44 45 ( Table 5 ). Besides anatomical delineation, fracture detection and osseous destruction in tumors can be sufficiently achieved by ZTE and Star-VIBE. However, despite recent development they still provide a slightly lower spatial resolution than CT ( Fig. 3 , Fig. 4 , Fig. 5 ). This can lead to oversight of microfractures, particularly linear nondisplaced fractures <1mm. One positive note is that MRI provides more information about microhemorrhages and other intracranial post traumatic findings, which play a pivotal role in therapy management. As a result, an MRI protocol including a dedicated bone sequence might score even better than CT in trauma settings. Dremmen confirms this approach using PETRA yielding higher sensitivity (100% vs. 81%), specificity (100% vs. 83%), PPV (100% vs. 94%), and NPV (100% vs. 55%) compared to CT for the simultaneous detection of fracture and intracranial hemorrhage 10 ( Table 3 ). While the MRI-based first-line approach is feasible in emergency departments that have a scanner at their disposal in the majority of the institutions, the logistical path will hinder its implementation. Furthermore, the patient's characteristics, such as age and metal implants, must be considered.

In comparison to ZTE in skull imaging, the FRACTURE and UTE sequences yield slightly inferior fracture delineation compared to CT, which is more pronounced in the FRACTURE sequence. This is confirmed by the group of Eva Deininger-Czermaka and explained by the main challenges of these techniques, namely the bone/air-interface, sutures, and intraosseous vessels 44 .

In the skull-base and the viscerocranium, air leads to signal drop-out and impairs bone-evaluation, making the bone/air interface difficult to distinguish. This effect is more pronounced in the Cartesian FRACTURE sequence and when the contrast is inverted to CT-like hyperintensity. Previous research has suggested solutions for this problem, such as complementing the magnitude images with phase information or segmentation with thresholding. Secondary features like hemorrhage and fluid accumulation in the mastoid cells and paranasal sinus make the cell walls and the fracture line visible. However, as these findings are frequently present in healthy subjects, careful evaluation is required.

In this regard a novel dual-radiofrequency and dual-echo UTE (DURANDE) achieves higher contrast in facial bones than ZTE, because it uses two types of RF-pulse with different lengths and two echoes for short and long T2* tissues, which enhance bone-structures after subtraction 11 ( Table 3 ). However, the differentiation of the cranial tabulae is better on ZTE due to the complete attenuation of bone marrow, while in UTE bone marrow still emits a signal. It can be concluded that novel multi-echo UTE-based approaches can be recommended for facial-bone imaging, while ZTE excels in cranial-vault delineation.

Apart from pneumatized areas, another limitation in the skull area are sutures that can mimic or mask undisplaced fractures in children younger than 2 years 43 . In this regard, it is advisable to use a 3D sequence, as it aids in the differentiation between fractures and vascular transosseous channels or sutures (increases the sensitivity to 83% and the specificity to 100%).

In nontrauma patients, black bone MRI aids in the evaluation of premature craniosynostosis, as reported by Lu and Low, and in conjunction with the conventional sequences, both cranial vault and intracranial anomalies can be depicted, as craniosynostosis often occurs as part of a multisystem syndrome. Lu has demonstrated the effectiveness of the ZTE in detecting various syndromal conditions. Cortical bone appears as a signal void while sutures and soft tissues exhibit intermediate signal intensities, which improves the distinction between patent sutures (high signal) and fused sutures (signal void) 45 . Craniosynostosis can also be depicted accurately with a non-Cartesian VIBE sequence using a golden-angle acquisition scheme (GA-VIBE) – a type of Star-VIBE 12 ( Table 3 and Table 8 ). The sequence is known for its motion robustness and improved bone/soft tissue contrast, and it achieves specificity and sensitivity of >95%. It employs an azimuthal angle of 111.25°, derived from the golden ratio, to achieve uniform radial coverage in k-space. Compared to ZTE-PETRA, it is less sensitive to motion, because the PETRA fills the center of k-space in a Cartesian manner. Therefore, for patients without sedation, it is advisable to use GA-VIBE or another type of ZTE, such as HyFi, if available.

Besides traumatic and developmental pathologies of the skull, tumors can also be accurately depicted. ZTE proved to be effective in evaluating lesions such as fibrous dysplasia, Langerhans cell histiocytosis, and retinoblastoma with good delineation of osseous destruction 4 .

A well-known issue of GRE is their proneness to susceptibility. However, in the skull region black bone MRI succeeds in effectively evaluating implant position, such as shunt catheter and orthopedic hardware, and differentiating between foreign bodies (gunshot injury) and bone fractures, especially using ZTE and FRACTURE. However, specific considerations must be met such as lower magnetic field and proper shimming 45 .

In summary, one should use ZTE in fracture detection of the cranial-vault and multi-echo-UTE in the skull base and viscerocranium. When it comes to craniosynostosis, it is better to use a Star-VIBE sequence or fully non-Cartesian ZTE. The performance of all sequences can be augmented by deep learning techniques, especially the combination of ZTE-based acquisition with a U-Net neural network.

## Spine

Moving forward to the spine region, black bone MRI has been extensively examined in traumatic, degenerative, and neoplastic conditions. In fracture detection, VIBE and ZTE yield great results with similar sensitivity (>90%) and specificity (>90%) to CT, which are, however, anatomy-dependent 8 15 46 47 55 ( Table 8 ). Compression fractures are more accurately depicted with confident determination of the fracture age than pars articularis fractures, particularly when the latter is incomplete (96.7% sensitivity and 92% specificity). This is due to the anatomy and the inferior spatial resolution of the sequences, which in the case of finer obliquely oriented structures in the pars interarticularis, lead to underestimation of the fracture lines or overestimation when sclerosis is present. To overcome these issues and enhance bone contrast, ZTE uses strong soft tissue suppression, whereas VIBE utilizes the Dixon-subtraction technique, allowing for selective enhancement of specific tissues of interest. A combination of both methods is possible using 3D stack-of-stars UTE with Dixon reconstruction, as demonstrated by Feuerriegel 14 . This fat-suppressed bone-sensitive sequence has the potential to replace the STIR sequence (using a water-separated sequence), T1 sequence (using a fat-separated sequence) and CT (using SWI phase masks applied to the magnitude image) ( Table 3 ). The water separated sequence detects bone marrow edema in acute fractures and SWI accurately detects fracture lines similar to CT with a substantial agreement assessed with weighted Cohen’s κ of 0.90 (Genant classification) and 0.75 (AO/Magrl classification). However, caution is necessary when utilizing the UTE sequence, as several modifications are required to enhance its robustness. 3D-UTE with slab selection using a soft, half RF-pulse outperforms the single-echo 3D stack-of-stars approach due to less pulsation and motion artifacts 50 56 . Using the slab-selective version in the coronal plane with a limited field of view (FOV) helps mitigate motion artifacts.

When it comes to tumor detection BoneMRI can aid the conventional technique by depicting cortical involvement showing features, such as cortical bone thinning, destruction, and periosteal reactions. In particular, the VIBE and ZTE sequences have demonstrated promising results in evaluating lesions in the spine, pelvis, and lower extremities, including those associated with multiple myeloma, fibrous dysplasia, giant cell tumors, and metastases 16 46 47 48 55 ( Table 8 ). Both sequences yield high accuracy compared to CT (98%); however, due to the sparse signal coming from bone, the MRI-derived HU values are generally lower than those from CT, an issue that can be alleviated with improved background signal suppression. While both sequences are effective for detecting fibrous dysplasia, VIBE tends to perform better for multiple myeloma by producing fewer false negatives. The false negative rate in ZTE can be explained by the reduced SNR and CNR and partial volume effects that come from limited coil coverage and the very low flip angle. Deep learning techniques can counter these issues in the future. Besides traumatic, degenerative, and neoplastic lesions, black bone MRI and sCT have been studied in the detection of inflammatory and congenital conditions.

In evaluating sacroiliac joint and lumbosacral junction anomalies in healthy children, sCT matches CT with perfect agreement on the presence of bony bridges (kappa 1) and good to excellent agreement on iliosacral anomalies, fusion, facet defects, and ossified nuclei (kappa 0.615–1). In cases of sacroiliitis, sCT effectively detects erosions and sclerosis and can even outperform low-dose CT, which is commonly used for pediatric patients (intrareader k 0.70–0.88 for sCT vs. 0.77–0.90 for CT; interreader k 0.70–0.90 for sCT vs. 0.75–0.84 for CT).

For adults, synthetic CT scans reconstructed from 3D-T1-RF-spoiled MGRE or ZTE provide higher diagnostic accuracy and reliability in detecting sacroiliac joint lesions compared to T1-weighted MRI/VIBE, achieving similar reliability to conventional CT (94% vs. 84% accuracy, 94% vs. 45% sensitivity, 96% vs. 89% specificity) 39 ( Table 3 ). ZTE alone surpasses VIBE in detecting erosions and sclerosis, offering higher sensitivity, specificity, and accuracy, while both sequences perform equally well in assessing joint width 57 .

In summary fractures can be successfully detected using VIBE, ZTE, and UTE, although pars articularis fractures and degenerative changes pose challenges due to anatomical positioning and bone mimickers. When available, slab-selective 3D UTE provides a broad range of tissue contrast, potentially replacing conventional sequences like STIR and T1. Tumors, degenerative conditions, and congenital anomalies are well-visualized with ZTE and even better with ZTE-based U-NET 19 .

Other specific applications of the black bone sequences include preoperative imaging, arthrography, quantification of degenerative and traumatic changes, age determination and dynamic joint imaging.

## Perioperative imaging

In preoperative planning and intraoperative guidance, bone MRI has been used successfully in the facial region and the spine 23 51 52 53 58 59 60 ( Table 8 ). 3D models were printed and compared with CT, which in most cases showed minimal deviations. However, the edge of the FOV and tendon attachments limited the image quality due to signal distortion and poor discernibility.

In preoperative planning and navigation of screw placements in the lumbar spine, ZTE and sCT present minimal deviation compared to CT (ZTE: median deviation 0.24 mm-0.27 mm, sCT mean absolute difference of 0.26 ± 0.24 mm). However, MRI-to-CT translation is more computationally and logistically demanding than directly using a bone-sensitive sequence 59 .

Synthesized CT from 3D T1-MPGR with patch-based U-NET visualizes normal and pathological osseous anatomy and estimates pedicle screw trajectories similar to CT, based on visual inspection 18 . The favorable aspect of this approach relies on the vastly available sequence used as a basis of the CNN and its excellent conversion into HU. The multiple-echo acquisition means that in-phase and opp-phase at different TEs are acquired, which provide insight into the effect of T2* decay and thus give more information about the tissue properties like water friction and fat fraction, susceptibility, and proton density. The in-phase outperforms the opposed phase due to the signal loss on water/soft tissue boundaries of the latter that cannot relate to the CT images adequately, but the most accurate sCT is generated from multichannel input. However, the MRI-to-CT translation can introduce inaccuracy, as some of the soft tissue contrast can get lost in the transfer. For simplicity purposes, bone-sensitive sequences can be used directly for screw guidance as the team of Franziska C.S. Altorfer has shown 53 ( Table 8 ).

Two black bone sequences, ZTE and SPGR, were merged with fluoroscopic images and then registered in a robotic software to mark the trajectories of the robotic arm in pedicle screw placement. They achieved sufficient accuracy in planning the screw-placement with median deviation of overall 0.25 mm. Both sequences were able to capture bone signal sufficiently after applying postprocessing to invert the contrast and mask the air. The SPGR sequence yields higher SNR and CNR than ZTE, but it is worse in bone/ligament differentiation and is more sensitive to motion. In the operative setting, breathing can cause signal distortion in the lumbar region, so it is preferable to use the ZTE sequence in this case.

ZTE can also be used in the preoperative planning in transcranial surgeries after being fused with MR angiography, while VIBE and 3D-T1-FFE depict bone fractures and tumor involvement in the viscerocranium 51 52 58 ( Table 8 ).

In transcranial surgery, fused ZTE/MRA images assist in planning procedures such as endarterectomy, aneurysm repair, and tumor resection by providing a clear visualization of the relationship between lesions and adjacent bone structures. For instance, the relationship between an ACOM aneurysm and the planum sphenoidale or between the ICA and the anterior clinoid process can be assessed, as well as the proximity of a tumor to cranial bones and cortical veins. In a preoperative setting not only simultaneous bone/vascular imaging, but also bone/nerve imaging is essential 60 .

In oral surgery a combined protocol of black bone (T1 FFE/VIBE) and fluid-sensitive (STIR/DESS) sequences provide information about osteolysis and damage of the lingual and inferior alveolar nerves, which is relevant, for example, in third molar extraction, periodontal bone resection and augmentation, and 3D-model generation 51 52 58 . T1 FFE visualizes bone pathologies similar to CT and the VIBE-based printed model compares to CT with a mean deviation of only 0.50 mm.

The DESS sequence effectively highlights nerve edema and engorgement due to its dual-echo approach. Due to its susceptibility sensitivity, the FID signal renders bone relatively dark, while the echo signal enhances fluid brightness, which allows for clear delineation of nerve structures against surrounding tissues 52 58 ( Table 8 ).

Challenging regions in this area are the mental region and the mandibular angles. As with the preoperative 3D-MRI based models of the lower arm, tendon attachments disturb the segmentation of the coronoid process, as the temporalis and masseter muscles insert there. Besides these constraints, involuntary movements in this region due to eye movement and swallowing can also distort the image, a way to diminish their impact is by using a non-Cartesian grid, which is how Star VIBE works. It uses an in-plane stack-of-stars technique for reduction of motion effects during phase-encoding and oversampling of the center of k space, which leads to better anatomical delineation. This is particularly useful for uncooperative patients or in the lower neck, where breathing artifacts are encountered. The low SNR of this sequence can be mitigated by using a dedicated small surface coil.

In conclusion, for operative guidance in the spine region ZTE alone or sCT from a multichannel input yields the greatest accuracy with CT. In the facial region, T1-FFE and STAR-VIBE are the sequences of choice in bone visualization, particularly in mandibular fractures and osseous tumor involvement, while the DESS sequence depicts neuronal injury. For transcranial endarterectomy, ZTE/MRA-fusion is the ideal approach.

## Quantitative UTE

The UTE sequence gives insights not only into morphology but also into quantitative information about short T2-structures, which has been thoroughly investigated over the past 5 years 20 21 22 35 54 61 62 63 64 65 ( Table 6 ). To evaluate osteoporosis, the porosity index and suppression ratio are effective tools, while for osteochondrosis, mapping techniques such as T2*, T1, and Tρ mapping are commonly utilized, as shown in Table 6 . Additionally, these mapping techniques are effective for evaluating cartilage and meniscus in osteoarthritis, assessing tendon structure in tendinopathy, and examining ligaments following traumatic rupture, degeneration, or postoperative recovery.

In osteoporosis, a 3D Dual Echo Cones UTE technique with two echo times is employed with a TE of 0.032 ms for bound water and 2.2 ms for pore water and fat. The porosity index is calculated as the ratio of the pore water signal to the total signal from bone, but with this technique the bound water is not measured directly 20 21 35 ( Table 3 ). This can be made by using a 3D IR-UTE-Cones sequence, which applies an adiabatic inversion pulse to suppress signals from pore water and fat. This outputs the suppression index, defined as the ratio of the total bone signal to the bound water signal. Both the porosity index and suppression index show strong correlation with microCT with even higher resolution of 2 μm versus 6–9 μm for microCT, effectively distinguishing normal bone structure from osteopenia and osteoporosis. Additionally, these indices can assess the tensile mechanical properties of bone under stress. Interestingly the porosity index can be used not only in the context of bone loss but also in bone thickening in athletes. Intense exercise regimes lead to increasing density and decreasing porosity of the bones, but when the healthy limit is exceeded, they are prone to stress fractures. It is important to detect the precursor changes before an injury occurs.

In addition to the porosity index and suppression index osteoporosis can be measured using an adiabatic inversion recovery prepared with STAIR-UTE-Cones 22 ( Table 3 ). The long-T2* tissue signal is suppressed, and the bound water signal is captured such that collagen density and hydration are the output. The resulting score correlates strongly with standard BMD and T-Score. Apart from osteoporosis 3D UTE is used for quantification of osteochondrosis via different mapping techniques of the acquired signal from the intervertebral structures like T2* mapping with standard 3D UTE, T1 mapping with 3D adiabatic-IR-fat saturated-UTE and T1ρ(rho)-mapping with 3D adiabatic-UTE 22 ( Table 6 ). In degeneration states the T2*, T1ρ and T1 values of the nucleus pulposus decrease and the T2* value of annulus fibrosus increases. This is due to the compression and degeneration of the nucleus and the subsequent migration of the water molecules to the annulus fibrosus. Thanks to these mapping techniques, the degeneration of the disc can be captured in an early stage.

Despite their advantages, these techniques remain experimental due to challenges such as partial volume effects, motion artifacts, and susceptibility artifacts. Additionally, signal interference from surrounding soft tissue can reduce performance. Optimizing TR/TI combinations for enhanced pore water suppression requires further investigation.

Apart from UTE recent research focuses on ZTE-based quantification methods of osteomalacia and osteoporosis 66 67 . These techniques are still in the early stages of investigation, having been tested on animal femur bones at a higher magnetic field strength of 7T, and they are based on phosphorus or hydrogen quantification in both trabecular and cortical regions. As of 2024, the ZTE results have shown a strong correlation with CT and gravimetric measurements. Cortical bone mineralization density was found to be lower in cases of osteomalacia and unchanged in osteoporosis, while trabecular bone mineralization density behaved in an opposite manner. Additionally, in osteoporosis, there was an expansion of the cortical region, which was not observed in osteomalacia.

In conclusion, to quantify osteoporosis the porosity index and suppression ratio using dual echo and IR-UTE with cones trajectory should be used. Additional fat suppression yields more information about disc degeneration. ZTE is an emerging alternative that is still in the early phase of investigation on animal specimens.

## Limitations and challenges

Most studies on bone MRI were carried out on small subject groups of between 30–50 and rarely exceeding 100, which limits their generalizability 11 22 50 . Additionally, many studies rely on cadaver imaging, which does not translate fully to living patients 13 20 44 45 53 ( Table 8 ). Future research should focus on larger, multicenter trials involving diverse patient populations and pathological conditions to validate these techniques.

Dedicated gradient echo sequences still face challenges with susceptibility artifacts and lower resolution compared to CT 3 32 55 . Unlike spoiled GRE sequences, ultrashort echo sequences are not widely available, limiting their broader clinical adoption. Recent reports regarding the availability of the dedicated bone MRI sequences have shown that the ZTE sequence of GE and its equivalents from the other vendors has been commercialized and is vastly available as summarized in Table 9 , Table 10 . However, it is not universally available across all MRI systems and may necessitate additional software or collaboration with the vendor for implementation. Likewise, the UTE sequence and FRACTURE sequence are not standard offerings and are often available only on select systems and may require additional software or hardware configuration. For instance, Siemens Healthineers offers UTE capabilities on their high-field MRI systems, such as the MAGNETOM Terra.X 7T scanner, which is equipped with advanced technologies such as Ultra IQ and Deep Resolve. However, such systems are typically found in specialized research institutions rather than standard clinical settings ( Table 9 ). The availability of UTE sequences in Germany is currently limited to specialized institutions, and a small number of these are mentioned in Table 11 . For patients or professionals interested in UTE MRI, it is recommended to inquire directly with university hospitals or major research centers. A widespread introduction into routine clinical practice has not yet taken place.

**Table:** **Table 9** Availability of black bone sequences.

Sequence Type	Siemens	GE Healthcare	Philips Healthcare
ZTE	PETRA commercially available on Siemens MRI systems equipped with the Quiet Suite package	Commercially available on certain systems (SIGNA Premier)*	Available in research settings
FRACTURE	Alternative sequences available (e.g. VIBE)	Alternative sequences available (e.g. LAVA)	Commercially available on certain systems
UTE	Available on select 3T systems, mainly in research or specialized clinical settings, with additional software (MAGNETOM Prisma, Vida, and Lumina)**	Available on some high-end systems, often requiring research collaboration or custom setup (SIGNA Premier and SIGNA Architect) ***	Available in research settings; not widely deployed clinically (Ingenia Elition and MR 7700)**
*do not necessarily require hardware modifications **may require additional software packages or custom sequence development ***may necessitate collaboration with GE's research and development teams or participation in specialized programs			

**Table:** **Table 10** .

Vendor	Notes on sequence acquisition
**Siemens**	Offers sequences as part of their research or clinical packages; sometimes available under “Works-in-Progress” (WIP) agreements, requiring institutional research approval.
**GE**	Offers commercial ZTE packages; UTE may require special software keys; costs can include licensing and system adaptation fees.

**Table:** **Table 11** Availability in Germany (sample selection).

Institution	Location	Sequences Available	Notes
**University Hospital Jena**	Jena	UTE	Engaged in developing and applying UTE sequences for imaging compact tissues such as tendons and ligaments.
**University Hospital Essen**	Essen	UTE	Participated in pilot projects for standardizing MRI diagnostics in multiple sclerosis, indicating use of advanced MRI techniques.
**University Hospital Heidelberg**	Heidelberg	FRACTURE	Conducted studies evaluating the diagnostic performance of the FRACTURE sequence for detecting and classifying proximal tibial fractures.
**University Hospital Freiburg**	Freiburg	ZTE	Involved in research assessing the utility of ZTE imaging for evaluating bony lesions, suggesting integration into standard care protocols.
**Technical University of Munich**	Munich	UTE	Assessment of vertebral disorders

The same applies to AI and deep learning techniques, which, despite their gradual integration into clinical practice, remain underutilized due to the limited availability of models, insufficient expertise, and poor generalizability resulting from constrained training datasets. To enhance the utility of AI models, larger, more diverse datasets and collaborative research initiatives are needed to refine model robustness and reliability. The lack of widespread expertise in advanced MRI techniques and deep-learning-driven image analysis remains a significant barrier. Addressing this issue will require educational initiatives, hands-on training programs, and simplified user interfaces for imaging software.

By addressing these limitations and incorporating emerging technologies such as AI and quantitative imaging, bone MRI techniques can evolve to become more reliable, accessible, and clinically impactful in routine and specialized care.

## Conclusion

In conclusion, assessing bone morphology with MRI is a complex yet valuable activity. The presence of tightly bound water molecules in bone poses a challenge due to the rapid signal decay, which can be mitigated using specialized sequences such as FRACTURE, UTE, ZTE, and VIBE. Alternatively, machine learning algorithms can generate CT-like images from MRI data. The selection of the most suitable method depends on clinical requirements, patient-specific factors, and technical or logistical constraints. As of 2025, the use of specialized preprocessing and postprocessing techniques remains essential to ensure optimal sequence performance, paving the way for radiation-free bone imaging. With ongoing advances in artificial intelligence, integrated post-processing software has the potential to streamline workflows, although its accessibility remains restricted to certain geographic regions.

Appendix: Basic principles of gradient echo sequences and black bone sequences

Gradient echo (GRE) sequences were developed as an alternative to spin echo (SE) sequences in order to reduce scan time; they employ a single RF pulse (<90°) combined with dephasing and rephasing gradients in the readout (frequency encoding) direction 68 . This approach creates echoes without relying on the 180° pulse, which makes them faster but also more sensitive to magnetic field inhomogeneities, leading to T2* weighting (combining T2 and static field effects). There are three basic types of GRE – spoiled, refocused unbalanced, and refocused balanced. The first employs shorter TR, TE, and larger flip angles (α) to achieve T1 weighting and utilizes RF spoiling to eliminate residual transverse magnetization through phase dispersion. Unbalanced refocused sequences allow partial rephasing and dephasing of the transverse magnetization, using gradient spoiling with constant gradients. Balanced refocused sequences implement phase cycling and precise refocusing gradients along the phase-encoding direction for optimal preservation of phase coherence and minimization of signal loss due to dephasing 69 .

The black bone sequences are modified spoiled gradient echo sequences that are able to capture the T2* signal of bones. The terminology surrounding “black bone” imaging is inconsistent across the literature. In some sources the term is reserved for conventional sequences, predominantly spoiled sequences such as SPGR, VIBE, but also FFE and FLASH, while others include the dedicated bone sequences with short TE: UTE and ZTE ( Table 1 ). The controversy stems from differences in the technique and the resulting image properties. The conventional sequences are unable to capture the signal coming from bone and other structures with tightly bound water, due to their fast T2* decay, hence the name black. In contrast the ultra-short TE sequences detect small amounts of signal coming from bones due to their quick transmit-receive switch. However, the captured signal is still less than that coming from the surrounding soft tissue, so qualitatively it appears darker (black) to viewers of the image. Apart from having a very short TE, they also use a small flip angle that suppresses signals from both fat and water, producing images with reduced contrast between soft tissues and resulting in bone appearing black while soft tissues occupy a narrow range of gray values. This technique improves bone representation and after appropriate post-processing, further enhancements in resolution and signal-to-noise ratio (SNR) can be achieved ( Fig. 2 ).
